# Crowdsourcing a Training Dataset of Question-and-Answer Pairs for AI-Enabled Health Information Tools on Sexually Transmitted Infections: Protocol for a Cross-Sectional Exploratory Survey Study

**DOI:** 10.2196/70005

**Published:** 2025-09-09

**Authors:** Elizabeth Oseku, Petra Kerubo Mariaria, Henry Semakula, Clare Allelua Kahuma, Martin Balaba, Agnes Bwanika Naggirinya, Rachel Lisa King, Rosalind Parkes-Ratanshi

**Affiliations:** 1 Academy for Health Innovation Uganda Infectious Diseases Institute Makerere University Kampala Uganda; 2 Department of Epidemiology and Biostatistics, Institute for Global Health Sciences University of California San Fransisco, CA United States; 3 School of Medicine, Dentistry and Biomedical Sciences Queen's University Belfast Belfast Ireland

**Keywords:** sexually transmitted infections, artificial intelligence, AI, health information, dataset, crowdsourcing

## Abstract

**Background:**

Sexually transmitted infections are a significant public health concern, particularly in sub-Saharan Africa, where their prevalence remains high. Promoting awareness and reducing stigma are essential strategies for addressing this challenge, but those affected often have limited access to accurate and culturally appropriate health information. Therefore, innovative solutions are essential to enhance sexual health literacy and encourage informed health-seeking behaviors. Artificial intelligence (AI)–enabled tools, such as chatbots, have emerged as promising avenues for delivering accurate and accessible health information. However, their potential is constrained by the lack of contextualized datasets, which are crucial for ensuring their effectiveness and relevance to diverse populations.

**Objective:**

This study aims to develop an open access, contextualized dataset of question-and-answer pairs on sexual health and sexually transmitted infections to support the development and training of digital and AI-enabled health information tools.

**Methods:**

Using a crowdsourcing approach, questions are being collected from participants aged ≥15 years via online platforms, paper-based submissions, and in-person interactions at public events across sub-Saharan Africa. Each question will be anonymized and reviewed by medical professionals who will provide accurate, evidence-based answers. The dataset will then undergo processing, including cleaning and tagging for AI training, ensuring adherence to findability, accessibility, interoperability, and reusability principles. The final dataset will be published as open access.

**Results:**

Data collection began on June 12, 2024, and is ongoing. The data collection process was piloted in Kigali, Rwanda, where 132 questions were collected. As of August 2025, the study had collected over 5620 question-and-answer pairs. The collected data are undergoing a simultaneous rigorous data processing phase in collaboration with health workers who provide evidence-based answers to the questions and new questions based on their experience in the clinic. The data cleaning and processing will enhance the utility of the data for AI applications.

**Conclusions:**

The final dataset will be published as open access in 2025, contributing to the development of AI-driven health tools and promoting public health literacy.

**International Registered Report Identifier (IRRID):**

DERR1-10.2196/70005

## Introduction

### Background

The burden of sexually transmitted infections (STIs) in sub-Saharan Africa (SSA) is high and increasing [1,2], accounting for approximately 40% of the global burden, with an incidence rate of 241 per 1000 among adults aged 15 to 49 years [3,4]. Challenges such as lack of awareness, stigma surrounding STIs, and poor access to medical care contribute to the high prevalence of STIs. As a key strategic action for primary prevention of STIs, the World Health Organization (WHO) recommends the provision of comprehensive, accurate, and culturally relevant information and education that promote sexual health and well-being [5]. Information, education, and counseling are effective approaches to improve health-seeking behavior in the context of STIs because people become better able to recognize the signs and symptoms of disease [6].

In recent years, internet access in Africa has been expanding rapidly due to improvements in infrastructure, including increased electricity availability, and the widespread adoption of digital technologies. The continent has seen significant growth in both internet connectivity and mobile phone use, creating opportunities for data-driven strategies and innovations [7]. This increasing access to the internet, combined with the rise of social media platforms, offers an opportunity to use these digital platforms to disseminate information about STIs on a large scale. With increased internet penetration, health care workers are more easily able to access technical STI resources [8], such as the Centers for Disease Control and Prevention, WHO, American Congress of Obstetricians and Gynecologists, and the Joint United Nations Programme on HIV/AIDS. When using the internet, the public, particularly adolescents and young adults, accesses STI information via social media platforms [9,10]. However, the information on these platforms may not be credible and may not have an adequate level of detail and risks, perpetuating myths and misconceptions about the subject [9,10].

Chatbots can understand and interact with human language through natural language processing, a branch of computer science and artificial intelligence (AI) that uses machine learning to help computers understand and communicate using human language [11]. There is emerging evidence that they are acceptable for use in public health. For example, a mixed methods study comprising interviews and surveys, conducted by Chang et al [12] in central Taiwan, found that users’ attitudes and subjective norms were significantly and positively associated with their intentions to use medical chatbots. Their study pointed out that these conversational agents are viewed more favorably when they are perceived as reliable, and much clinical work is itself quite conversational. Their study also suggested that the use of chatbots was acceptable due to their accessibility and anonymity [12]. In addition, the study by Miles et al [13], conducted among adults in the United Kingdom, suggested that chatbot acceptability might be higher for stigmatized health issues. For illnesses that have a high level of perceived stigma, such as STIs, chatbots may offer greater anonymity than face-to-face consultations. This was highlighted by an increased willingness to disclose sensitive health information to chatbots in comparison to health care workers [13]. Research also shows that medical practitioners recommend chatbot use for the provision of medical information. In the United States, a study involving 100 general practitioners showed that more than half of the physicians (54%) agreed that health chatbots could help patients better manage their health and improve access to and timeliness of care [14].

### Comparison With Prior Work

A review of 12 studies by Phiri and Munoriyarwa [15] emphasizes the opportunity that health chatbots present in Africa for making health information more accessible. Nonetheless, they and other authors frequently point to a common limitation that holds back this promise—not having enough quantity or quality of contextualized data to train the chatbots. Chatbots need to be trained with a knowledge base relevant to the subject area so that they can adequately respond to the queries of users. However, access to large datasets that have been adapted to the diverse linguistic, cultural, epidemiological, and socioeconomic realities of the African continent is a challenge [15,16]. DataKind UK [16] discusses the importance of “decolonizing” the data that AI is trained on so that it reflects the lived experiences of groups that are underrepresented in both the health and technology sectors. These datasets must include question and answer (Q&A) pairs that cover topics relevant to the potential users, accounting for factors such as local languages, including slang and colloquial expressions, particularly about sensitive subjects, such as sexual reproductive health (SRH). Generally, the terminology associated with sex and SRH tends to use population-specific words that change frequently and vary by age group, location, education level, and other social factors, with people often using it to discuss private or intimate matters, such as STIs. For example, male genitalia are generally referred to by different names such as penis, kettle [17], whopper [18], bazuka [19], etc in the south, east, and west African regions, respectively. The training datasets should also adapt to the users’ varying educational levels and lived experiences.

The socioeconomic realities of the African continent mean that questions and advice on health care access may be different from higher-income settings. For example, in most resource-limited settings, STIs are treated through syndromic management. It is rare to undergo testing for individual STIs. Therefore, datasets must seek to give responses that balance what is on the ground in resource-limited settings as well as give information about best or newer practices that may exist elsewhere in high-income economies. This will ensure that the chatbots offer accessible, accurate, and empathetic responses.

### Rationale

To maximize applicability in the African setting, we propose crowdsourcing from populations across Africa. By using crowdsourcing, as recommended by Abhigna et al [20] and the Implementation Research and Innovation Support [21], this study ensures the inclusion of genuine, community-specific concerns, capturing the diverse linguistic and contextual nuances. Crowdsourcing is a shared computing method that taps into the collective knowledge and skills of people to solve problems that are difficult for computers but easily handled by humans, such as labeling data, speech recognition, and software development [22]. This approach has been found to be beneficial in AI because it enables the collection of large amounts of diverse data within a short period, which reduces bias and increases data richness [20,23].

While many datasets used by large language models or generative AI systems are not formally verified by medical professionals, the dataset we aim to construct will be carefully curated and validated by health care professionals to ensure its medical accuracy and reliability. This is crucial when dealing with sensitive topics, such as sexual health and STIs, where incorrect or misleading information could have serious consequences [24]. Crowdsourcing will allow us to gather authentic insights directly from the populations most affected [21], and by combining these crowdsourced data with expert validation, we ensure that the chatbot delivers accurate, medically sound information.

### Study Objectives and End Points

#### Overall Aim

This study’s general aim is to engage a wide range of people from across Africa to collect relevant context-specific questions about sexual health and use evidence-based medical knowledge to develop answers to the questions. Therefore, this will produce an open access, contextualized Q&A pair dataset on sexual health in English, which can be used to train AI-enabled health information tools.

#### Specific Objectives

The specific objectives of this study are as follows:

1. To collect at least 5000 contextualized English language questions on sexual health and STIs from the public through crowdsourcing via the internet and public events in SSA over a period of 6 months
2. To provide at least 2 accurate, evidence-based answer formats, based on the WHO guidelines, to each question collected on sexual health and STIs from the public in English in SSA
3. To process and curate the pairs into a training dataset for AI-enabled sexual health information tools
4. To provide the public with an open access, contextualized training dataset of Q&A pairs on sexual health and STIs in English

The dataset will be made available with a framework for adapting to different languages and contexts to be used in different geographic and sociodemographic settings across the continent.

## Methods

### Study Setting

This study will be conducted as part of a network activity for the Hub for Artificial Intelligence in Maternal, Sexual and Reproductive Health (HASH) [25]. As of January 2023, the HASH network is composed of 10 subgrantees from 7 African countries. These subgrantees are students, start-ups, and established organizations that won grants from HASH to research and develop AI innovations through a competitive request for applications. In total, 3 of these subgrantees are developing chatbots to relay information about sexual health, in particular STIs, in different settings in Ethiopia, Kenya, and Nigeria.

### Study Design

This study is a cross-sectional exploratory survey.

### Screening and Enrollment

There are various methods for recruiting participants in this study. Data collection will be done through crowdsourcing from members of the public, including but not limited to students, colleagues, and professionals aged ≥15 years from different locations and using different methods to ensure diversity in responses.

First, invitations for participants to contribute to crowdsourcing will be extended through advertisements via the internet media. The link to the online platform will be shared widely on the social media platforms of HASH and its partners and networks to attract as many participants and questions as possible.

Second, invitations will be extended through signposts and word of mouth at physical gatherings, such as relevant health or AI conferences, meetings, and other public events or locations. At physical public events or locations, a designated area with physicians will be set up for question collection and advertised by signposting and word of mouth. A link to the online platform will also be provided at the physical locations. For an informative and robust dataset, Q&A sessions from previously held SRH conferences will be added.

### Participant Selection

The eligibility criteria designed to select participants for whom protocol treatment is considered appropriate are presented in Textbox 1.

**Inclusion and exclusion criteria.**

**Inclusion criteria**

- Must be aged ≥15 years
- Must be able to speak, write, and comprehend English
- Must be willing to give consent or assent for their questions on sexual health to be anonymously discussed, responded to, and shared publicly
- Must be willing and able to comply with the determined modes of crowdsourcing
- Evidence of personally signed informed consent or assent in the case of minors, indicating that the participant (or a legal representative) has been informed about all pertinent aspects of the study

**Exclusion criteria**

- People who are unable to read and write due to disability
- People with mental impairment or those experiencing serious health issues

The team will deploy various measures to ensure that only eligible participants will enroll in the study. First, data collection sites for physical recruitment will be at locations and gatherings where we are certain that there will be no participants aged <15 years and where the language of conduct is English (eg, academic conferences or English language workshops). Any non-English language questions were excluded from the dataset. In addition, while the link to the online platform was shared widely, even on forums that might include those outside our target population, the online consent form asked participants twice to confirm that they were aged >15 years.

There were no specific exclusion criteria or questions to determine the location and nationality of the contributor, as the recruitment strategies were designed to maximize African participation; however, if there was a small proportion of questions from non-African individuals, given that STIs are a global issue, we did not deem this to be a problem for the study.

### Study Procedures

#### Overview

Participants will submit questions related to any aspect of sexual health, particularly STIs, through 3 modes: an online platform developed on Slido (Cisco) [26], a widely used tool that allows anonymous question submission and feedback; anonymous question submission on paper; or face-to-face interaction with a physician. A web link to the online platform will be shared with online communities for their submission.

**Figure 1:**
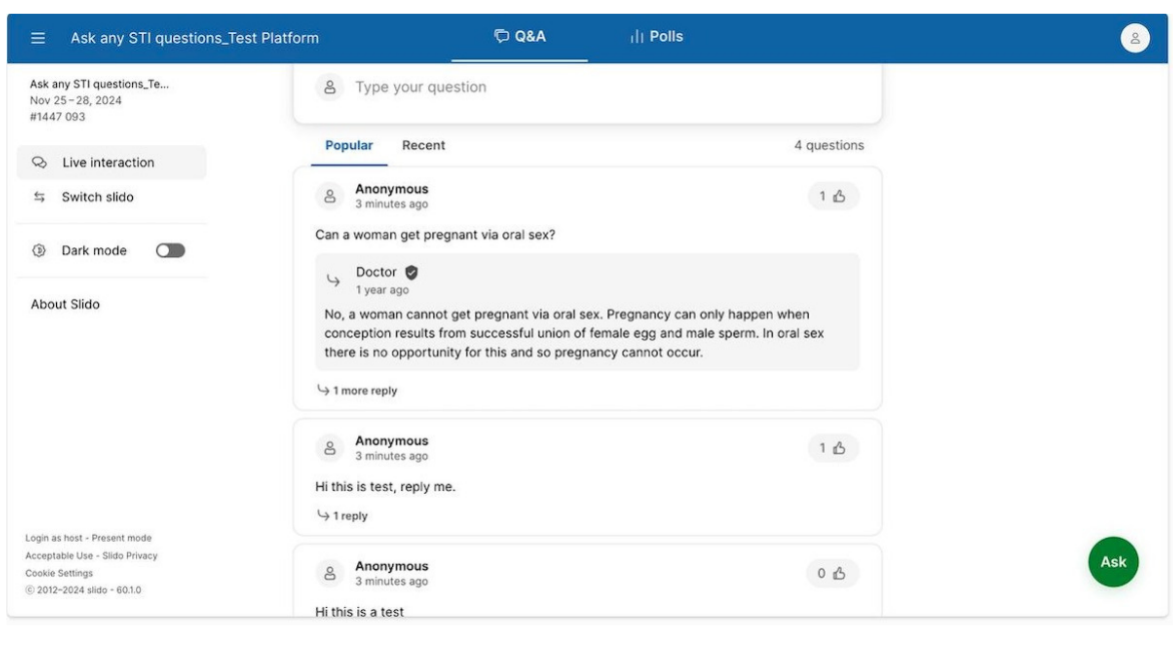
Slido interface.

At physical public events or locations, a designated area will be set up for question collection, and a link to the online platform will also be provided. A physician will be available to provide accurate answers to questions posed via the 3 modes. Figure 1 shows the Slido interface used for anonymous question submission and real-time interaction.

**Figure 2:**
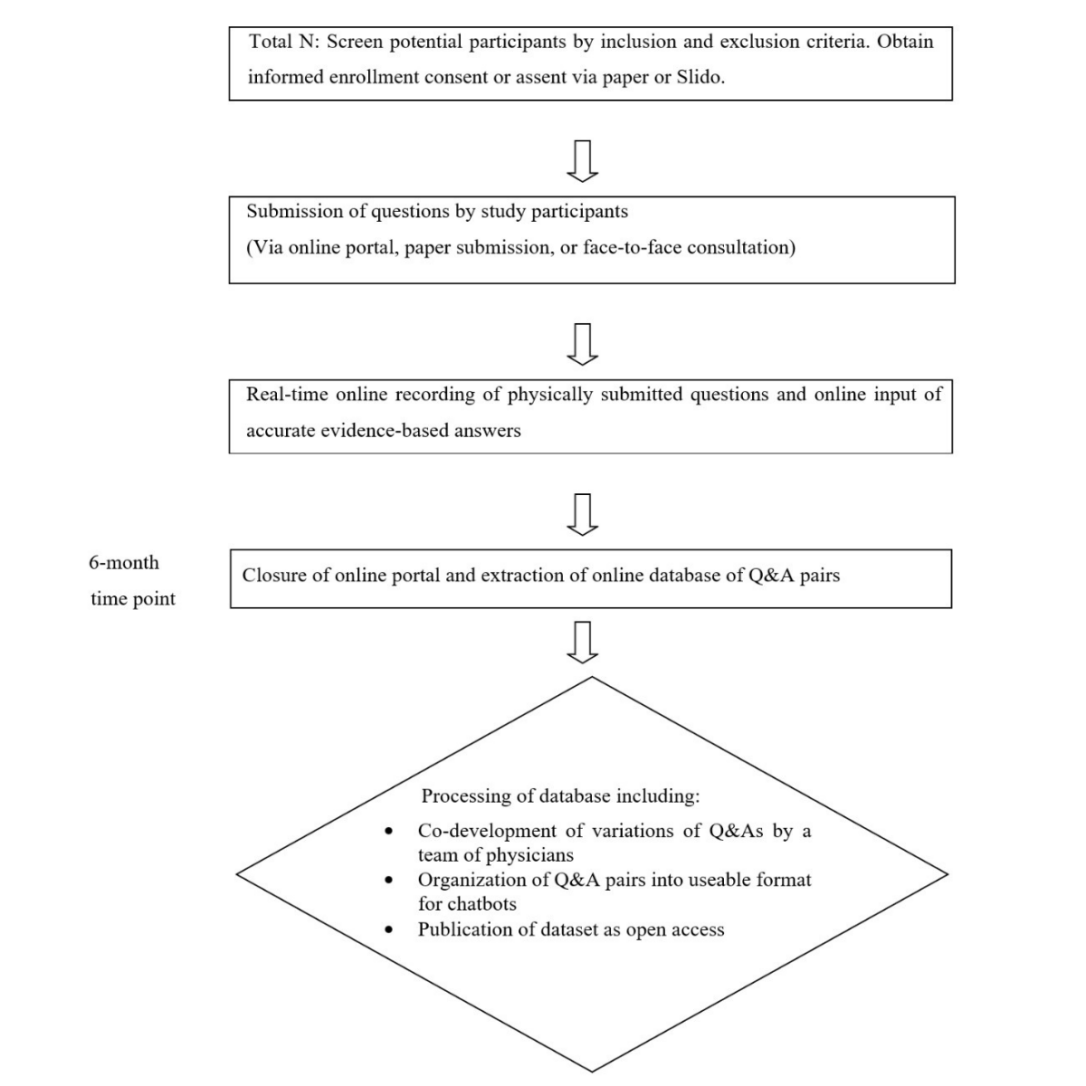
Schematic of study design. Q&A: question and answer.

Therefore, there will be no limit to the number of participants enrolled in the study to pose questions, how many questions a participant can pose, or the number of times a participant can engage to ask questions. All questions will be posed anonymously through the Slido platform, which ensures that the participants’ identities are not captured when submitting questions. Slido will assign each participant “Anonymous” as their name, so their personal details remain confidential, creating an environment for open and honest participation. Figure 2 illustrates the study design.

Study visits will be self-directed, allowing participants to engage with the data collection process at their convenience. After a participant provides informed consent or assent as the preliminary step, they are eligible to return as many times as they desire to their preferred data collection mode (ie, online platform, paper, or face-to-face consultation) to submit anonymous questions, check answers as provided by qualified health workers, or review other participants’ anonymous Q&As. For centralization and consistency, all questions collected outside of the online platform will also be recorded on the platform. To promote health literacy, participants will be able to view Q&As submitted by them or other participants and contribute to discussions through “replies,” which may stimulate further questions, enhancing learning. These “replies” are also an opportunity for clarification or to ask follow-up questions. This approach ensures that participants benefit not only from their own inquiries but also from the collective knowledge and experiences shared within the study.

There will be no follow-up of study participants, and all questions will be logged anonymously. From its launch, the online platform will be open to questions for 6 months. While participants will be able to interact with the online platform independently, there will be contact information on the platform and the informed consent and assent document through which participants can communicate with the study team in case of any challenges.

#### Withdrawal

Participants may withdraw from the study at any time at their own request, or they may be withdrawn at any time at the discretion of the investigator or sponsor for safety, behavioral reasons, or the inability of the participant to comply with the protocol-required procedures.

Because there are no follow-up visits, participant withdrawal will not necessitate an effort to contact the participant. The study team may retain and continue to use any data collected before such withdrawal of consent. However, should the participant wish to withdraw their question after submission, if they entered it directly onto the online platform, they are free to log back in and do so themselves. However, if their question was submitted on paper or through a face-to-face consultation, the participant should contact the study team and request withdrawal of their particular question.

### Assessment of Safety

Safety monitoring for this study will focus on unanticipated problems involving risks to participants, including unanticipated problems that meet the definition of a serious adverse event.

#### Unanticipated Problems

Unanticipated problems involving risks to participants are defined as any incident, experience, or outcome that meets all the following criteria:

- Unexpected in terms of nature, severity, or frequency given the research procedures that are described in the protocol-related documents, such as the institutional review board (IRB)–approved research protocol and informed consent or assent document, and the characteristics of the participant population being studied
- Related or possibly related to participation in the research (possibly related means there is a reasonable possibility that the incident, experience, or outcome may have been caused by a procedure involved in the research)
- Suggests that the research places participants or others at a greater risk of harm (including physical, psychological, economic, or social harm) than was previously known or recognized

#### Adverse Event Reporting

As this is an exploratory survey collecting anonymous questions about sexual health, we do not anticipate any adverse events.

Because this study does not test any investigational drug or intervention, and causality assessment is not applicable, any possible adverse events will also be reported to the IRB and the Uganda National Council of Science and Technology (UNCST) annually in aggregate.

The investigators will generate and submit annual reports summarizing these adverse events.

### Data Processing

There will be no fixed participant sample size, but the study will aim to create a dataset of 5000 English Q&A pairs. There is no universally accepted minimum sample size for training AI models; AI training does not typically use conventional power calculations, but sample size adequacy in this context is contingent upon various criteria, including task complexity, label consistency, and model architecture. According to empirical studies in machine learning, larger, high-quality datasets improve model accuracy and generalization. The selection of 5000 questions will provide a diverse dataset and is feasible considering the time and resources of this study.

All submitted questions will be reviewed and answered collaboratively by a team of at least 3 health workers using evidence-based information from reputable medical sources, such as the WHO, Centers for Disease Control and Prevention, Medscape, and UpToDate, guaranteeing medical accuracy. For information that may be country specific, the physicians shall source responses from official governmental or nationally recognized documents. We will aim to recruit health workers from various cadres, such as physicians, reproductive health nurses, and counselors, as their different experiences and expertise can enrich the quality of the answers provided. Each answer will be deliberated and jointly agreed upon before being entered into the dataset. This study provides sexual health information in real time to participants as a service; therefore, any question posed will be documented and answered, either face-to-face or on the online platform. For purposes of the final dataset, where participants submitted questions that are only partially related to STIs, the data processing team will include broad themes, for example, “safe sex practices,” “myths and misconceptions,” and “education”; the physicians will answer partially related questions and include these in the final dataset. Responses will be provided in real time to the participants for those questions determined by the data processing team to be completely unrelated to STIs, and the questions will also be documented in our source documents, but they will not be included in the final dataset.

The health workers will also work closely with a data scientist to ensure that the dataset is suitable for training chatbots. For a chatbot developed using this dataset to have a wide range of understanding, it is necessary for its knowledge base to be as comprehensive as possible in terms of the various ways questions may be asked and the answers to those different variations. For example, the team will create some additional variations of question format, and for each question, there will be both short and long (more detailed) answers to support different questions that could be encountered by the chatbot. Hence, questions with the same meaning but different wording will be included.

When data collection is complete, the database of Q&A from the online portal will be extracted and processed into a training dataset that can be used for the development of AI-enabled information tools such as chatbots. This process will include labeling each data entry with relevant tags, called intents (eg, prevention, treatment, and symptoms), to facilitate easy retrieval of information. Each individual question with its relevant short answer and the same question with its relevant long answer will be considered as 2 Q&A pairs. All similar Q&A pairs will be grouped under one intent. The dataset will be in JSON format [27] to support interoperability with existing health IT systems via Fast Healthcare Interoperability Resources standard [28] and as recommended by the findability, accessibility, interoperability, and reusability (FAIR) data principles [29]. Data cleaning and annotation will also be done using OpenRefine to speed up these processes and ensure high-quality results.

### Quality Control and Assurance

During the study, periodic monitoring will be conducted to ensure the study’s adherence to the protocol and Good Clinical Practice (GCP) guidelines. In addition, the study site may be subject to independent audits and inspections by appropriate regulatory authorities, such as the IRB, from time to time.

### Record Retention

The investigator has ultimate responsibility for the collection and reporting of all questions entered through the different data collection methods, that is, online, hard copy case report forms, and face-to-face consultations (source documents), and ensuring that they are accurate, authentic and original, attributable, complete, consistent, legible, timely (contemporaneous), enduring, and available when required. Any corrections to entries made in the source documents must be dated, initialized, and explained (if necessary) and should not obscure the original entry.

To enable evaluations and audits, the investigator agrees to keep records, including the identity of all participating patients (sufficient information to link records, eg, case report forms), all original signed informed consent or assent documents, copies of all safety reporting forms, source documents, detailed records of treatment disposition, and adequate documentation of relevant correspondence (eg, letters, meeting minutes, telephone calls, and reports).

Investigator records must be kept for as long as required by applicable local regulations (UNCST generally requires the records to be kept for a minimum of 5 years). When more than 1 requirement can be applied, records must be maintained for the longest period provided.

### Protocol Deviations

A protocol deviation is any noncompliance with the clinical study protocol, GCP, or the manual of procedures requirements. Noncompliance may be on the part of the participant, investigator, or study staff. Because of deviations, corrective actions are to be developed by the study staff and implemented promptly.

All deviations from the protocol must be addressed in study participant source documents and promptly reported to the local IRB according to their requirements.

### Ethical Considerations

Ethics approval has been obtained from the Infectious Diseases Institute Research Ethics Committee (2024-91) and the UNCST (HS5173ES). The study also obtained a waiver of parental consent for minors from the research ethics committee because even though the study posed minimal risk to participants, in the African cultural setting, parents may feel uncomfortable giving consent to their children being requested to ask questions regarding STIs. Therefore, participating minors will enroll with individual assent only.

This study will be conducted in accordance with legal and regulatory requirements as well as the general principles outlined in the International Ethical Guidelines for Biomedical Research Involving Human Participants [30] and the Declaration of Helsinki [31]. In addition, the study will be conducted as per the protocol, GCP guidelines, and applicable local regulatory requirements and laws. Participants will provide written informed consent or assent via online forms or hard copy forms. The record of consent or assent will be stored electronically for the online forms or via hard copy records for those participants who sign physically. All questions will be submitted anonymously, and parties will ensure the protection of participants’ personal data and will not include participants’ names on any forms, reports, publications, or disclosures, except where required legally. The informed consent or assent document used in this study and any changes made during the study will prospectively be approved by the IRB.

Participants will not receive any financial compensation for their participation, but they will benefit from receiving an accurate evidence-based answer to their question from a qualified health worker.

Data will be collected online, on paper, or through face-to-face consultations with physicians. Each participant will submit at least 1 question, and all questions will be recorded anonymously. Questions submitted via paper and face-to-face consultations will also be transcribed into the online platform for record purposes. Access to the online platform will be password protected and limited to only study staff with role-based access control (ie, admin, editor, and viewer) to ensure participant confidentiality and data integrity.

To protect privacy, both the online and physical consent forms advised study participants to carefully phrase their questions in a way that protected them and others. Where participants’ questions contained personally identifiable information, the study team had moderation rights through the Slido platform to make edits to the questions to ensure anonymity. Hard copy consent or assent forms, handwritten questions, and any other source documents will be kept in locked cabinets. The online platform’s database will be hosted in a secure cloud server owned by the Infectious Diseases Institute. Access to the database will be given to authorized personnel only (members of the immediate study team), and a log of authorized personnel will be stored in the trial master file.

### Publication of Study Results

When the dataset is completed, in adherence to the FAIR principles [29], we shall use Harvard Dataverse to host the dataset. Harvard Dataverse is a free, self-service data repository open to all researchers from any discipline, both within and outside of the Harvard community, where researchers can share, archive, cite, access, and explore research data. Each Dataverse collection is a customizable collection of datasets (or a web-based repository) for organizing, managing, and showcasing datasets [32].

## Results

### Overview

Piloting of the process was done at the AfricAI conference in Kigali, Rwanda, held in 2023 [33]. The conference targeted participants from Africa’s AI communities and ecosystems, but there are no specific demographic conference data. A QR code for Slido was generated and shared with participants at a booth and on fliers around the conference venue. Furthermore, the QR code was printed on small chocolates in the session rooms. These advertising efforts played a crucial role in raising awareness and expanding the reach of the pilot study, highlighting the value of sustained promotional strategies for maximizing participation. During this pilot, physicians were available on-site to answer questions in real time for those posing questions. The pilot study collected 132 questions from the conference over 3 days. Only 1 question was unclear and could not be answered. The physician responded on the online platform stating this. Another question was simply a greeting. All the remaining 130 questions were answered within 24 hours, and these were documented on the online portal. The question themes are categorized in Table 1.

**Table 1:** A table showing the categorization of questions collected through the pilot study (N=130).

Categorization of questions	Questions, n (%)
STI^a^ transmission	19 (14.6)
STI signs and symptoms	13 (10.0)
STI testing and diagnosis	6 (4.6)
STI treatment	9 (6.9)
STI complications	1 (0.8)
STI prevention	1 (0.8)
Contraception	14 (10.8)
Relationship advice	21 (16.2)
Circumcision	2 (1.5)
Pregnancy	9 (6.9)
Menstruation	9 (6.9)
Morality	4 (3.1)
SRH^b^ education	9 (6.9)
Vaccination	2 (1.5)
Other reproductive conditions	10 (7.7)
Menopause	1 (0.8)

^a^STI: sexually transmitted infection.

^b^SRH: sexual reproductive health.

### Integration of Pilot Lessons and Current Progress

The pilot study revealed the need for a large, supportive team to actively engage with participants, answer questions promptly, and monitor the process. This team-based approach ensured that participants felt encouraged and supported throughout, and professionals could consult internally. The importance of real-time monitoring and support during data collection was also evident, as it enabled the resolution of any challenges encountered and ensured the smooth execution of the pilot study. Following the pilot study, full recruitment and data collection for this study began in June 2024 and will continue for 30 months. As of August 2025, the study had collected more than 5620 Q&A pairs (including the 132 questions from the pilot phase). The collected data are simultaneously undergoing a rigorous processing phase, which involves cleaning and tagging to facilitate their use in training AI tools. The data have been grouped into 8 STI areas comprising the common STI topics: general STIs, HIV, syphilis, gonorrhea, chlamydia, hepatitis B, trichomoniasis, and herpes simplex virus. The questions have been categorized into key themes, such as prevention, treatment, symptoms, and other subcategories, to enhance usability for AI-enabled health information systems. In total, 11 workshops have been held with health workers of various cadres, including physicians, nurses, counselors, and pharmacists, to develop accurate answers for each STI topic and category and add questions to the dataset based on their experience in the clinic.

Upon completion, the dataset will be hosted on the Harvard Dataverse open access repository [32], with publication planned for mid-2025. This effort aims to create a valuable resource for AI developers and public health initiatives, particularly in the African context.

## Discussion

### Strengths

This study has several strengths that contribute to its innovation, reliability, and broader impact. It leverages an innovative crowdsourcing design to gather questions from a diverse and geographically dispersed participant pool, ensuring data diversity and relevance. This will allow the inclusion of real-world concerns, enhancing the cultural and contextual accuracy of the resulting dataset. The involvement of health workers of different cadres in the processing of the dataset will also ensure the medical accuracy of the answers to the questions collected. This aspect of collaborative expertise is a cornerstone of this study and will enhance the reliability and utility of the final dataset.

To maintain the accuracy and relevance over time, the dataset will undergo regular updates at least annually by a team of medical experts and a data scientist. Continuous feedback from participants and stakeholders will ensure that emerging topics and linguistic changes are also captured. This process will enable the dataset to evolve alongside medical knowledge and public health needs.

In addition, anonymity and privacy are emphasized, encouraging participants to share authentic and nuanced data on sensitive topics such as STIs without fear of judgment. The study will also publish its dataset as an open access resource, adhering to FAIR principles [29], which will support HASH subgrantees in their chatbot development and benefit the broader AI and public health community. Furthermore, this study offers a scalable model that can be adapted to other health domains or regions, leveraging the increasing internet penetration across Africa. Together, these strengths enhance the study’s potential impact and utility.

### Limitations

Despite its innovative approach, this study faces several limitations. Selection bias may arise as it excludes individuals without internet access, English literacy, or the confidence to ask questions about sexual health. In addition, this study does not have specific targets or sample size calculations for the different demographic groups. This could potentially limit the dataset’s representativeness for marginalized populations and lead to overrepresentation of certain demographic groups. However, setting targets for each population group may also limit participation because there was no way to know how many questions could come from an individual or section of the population, and even then, some questions cut across population groups. Therefore, we chose to keep the data collection open and seek opportunities to collect data from different populations (eg, universities; conferences; and clinical settings, such as rural and urban health facilities) and then consider saturation (where no new questions are being received) as an indicator that we have collected most available questions. Finally, the regular updates that the study team will continually provide to the published dataset will also serve to incorporate any new questions that emerge later.

There is also a risk of misinterpretation as some questions may not fully capture the participants’ intended concerns, leading to mismatched answers. This study’s anonymous nature, while ensuring privacy, limits opportunities for clarification. Maintaining the sustainability of the dataset in the long run might require consistent funding and collaboration with public health organizations, which is an ongoing consideration.

The exclusive focus on English further narrows the scope, overlooking the unique concerns of non-English speakers. Although strides have been made recently, there remains a significant absence of datasets that are fine-tuned to the many forms of African speech and the continent’s almost 2000 different languages. This is emphasized in studies by Javaid [23] and Ochieng and Awosiku [34]. The absence of such datasets in a key demographic region means that AI tools will perform comparatively poorly in this part of the world. This study aims to produce a training dataset for chatbots and other AI-enabled conversation agents. AI models require training before they can work, but this training requires a vast amount of data from multiple sources, including books, dictionaries, scientific papers, etc. At present, unfortunately, these resources are most commonly available in what are termed highly resourced languages, such as English, Chinese, French, etc. Most native African languages tend to be grouped under low-resourced languages, meaning they have relatively less data available for training AI models. However, the investigators are aware of the need to strengthen the presence of native African languages in the AI space.

The recruitment was done in Africa-based meetings or on African forums. While there may be non-African individuals accessing these events or online forums, due to the content of these forums, we can assume that they have significant knowledge of and interest in the African context. Therefore, their submitted questions would not introduce significant bias. Nonetheless, because of our emphasis on anonymity, this does remain an unintended weakness of our study.

Finally, the 6-month data collection window may not be sufficient to account for changes in public engagement over time, potentially affecting the comprehensiveness of the dataset. These limitations underscore areas for improvement in future iterations of the study.

### Potential Risks and Benefits

#### Risks

Overall, this study is considered to have minimal risk of injury to participants. In addition, this study has been designed to provide multiple options for participants to ask questions, maintain privacy and anonymity, and minimize embarrassment and stigma.

However, there is a minor risk of unintended psychological triggers related to abuse, stigma, or trauma that may occur as participants engage with our study. We provide real-time answers from health workers, and these answers can provide guidance on where to seek help for health-related issues that a participant may be facing related to a particular question. If psychological distress is identified by the health worker, then this will be addressed, and the participant will be guided on where to seek help. We anticipate that through the informed consent process, participants who may experience psychological distress will assess their risk, and this will minimize enrollment of participants who may be triggered by the study. Where participants do eventually enroll, the option to withdraw at any time of psychological distress is made clear at the informed consent stage. However, this assessment of risk is left to the discretion of the participant should psychological distress occur. We also have a disclaimer in our online portal reminding participants of the option to seek medical advice in their local setting.

This dataset is designed to train a chatbot, but the content and the chatbot design must be contextualized to the local population it will be used with. This includes adaptation to local expressions and cultural nuances and review of questions for cultural sensitivity. Therefore, any chatbots using this dataset must be validated before use, and training with this dataset does not replace validation of any AI tools that are trained on it.

#### Benefits

The dataset on sexual health that will be developed through this study will be used to enrich the training data of the HASH subgrantees who are developing chatbots. This will enhance the ability of their chatbots to respond to various queries that will be presented during interaction with end users and therefore support these members of the HASH network in making their research successful.

In addition, because the dataset will be open access, it will be made available to all users who desire a training dataset for their AI-enabled information tools on sexual health. This will contribute toward solving a significant need for African AI developers, which is for large datasets. It will also contribute toward combating misinformation and improving public health literacy in Africa by increasing access to accurate medical information.

Finally, during the crowdsourcing process, participants will receive accurate medical answers to their questions and have access to other Q&As gathered through the crowdsourcing process. This will improve the knowledge of participants.

In constructing an English STI dataset, we are seeking to produce a dataset to support the development of AI tools based on the resources we currently have. However, the plan is that the HASH project will develop a framework so that this dataset can be contextualized for specific populations, including translation into other African languages and adaptation with cultural nuances and sensitivities for each group of target beneficiaries. We aim to have a Swahili version and at least 1 other language version of the dataset by early 2026.

### Conclusions

This study represents a significant step toward developing accessible evidence-based health information tools, with the potential to increase literacy levels regarding STIs and improve health-seeking behaviors. The Q&A dataset from this study will enable the development of AI tools to address critical gaps in sexual health education, fostering informed decision-making. The open access nature of the dataset will encourage collaboration while providing a resource for researchers and developers worldwide.

The developed chatbots will need full contextualization, testing, and validation for the population they aim to serve. We also need to prioritize expanding linguistic diversity and accessibility of medical data, including evidence and guidelines, so that we can develop AI tools for underserved populations to ensure broader applicability and equity in health information dissemination.

## Abbreviations

AI: artificial intelligence
FAIR: findability, accessibility, interoperability, and reusability
GCP: Good Clinical Practice
HASH: Hub for Artificial Intelligence in Maternal, Sexual and Reproductive Health
IRB: institutional review board
Q&A: question and answer
SRH: sexual reproductive health
SSA: sub-Saharan Africa
STI: sexually transmitted infection
UNCST: Uganda National Council of Science and Technology
WHO: World Health Organization
